# Smartphone applications for informal caregivers of chronically ill patients: a scoping review

**DOI:** 10.1038/s41746-022-00567-z

**Published:** 2022-03-21

**Authors:** Milena Guessi Margarido, Amika Shah, Emily Seto

**Affiliations:** 1 Institute of Health Policy, Management and Evaluation, Dalla Lana School of Public Health, University of Toronto, Toronto, CA Canada; 2 Centre for Global eHealth Innovation, Techna Institute, University Health Network, Toronto, CA Canada; 3 Department of Computer Systems, Institute of Mathematics and Computer Science, University of São Paulo, São Carlos, Brazil

**Keywords:** Patient education, Quality of life

## Abstract

Mobile-health applications can be used to deliver timely and personalized health information to family and friends of chronically ill adults living in the community. This scoping review aims to investigate the nature and extent of native smartphone applications for informal caregivers. Six databases were searched for articles on applications across ten chronic conditions, namely heart disease, stroke, cancer, chronic obstructive respiratory disease, asthma, diabetes, Alzheimer’s disease or other dementia, rheumatoid arthritis, hypertension, and mood or anxiety disorders. In total, 36 articles were included, encompassing 26 applications. Of these, smartphone applications were designed for use only by caregivers (*n* = 15), with a few applications also intended to be used with patients (*n* = 5), healthcare providers (*n* = 4), or all three roles (*n* = 2). Most applications targeted a single chronic condition (*n* = 25), with Alzheimer’s and other dementia being the most common (*n* = 18). Only one application was designed for management of multiple chronic conditions. Long-term evaluation methods are needed to continually assess the impact of applications on a range of process and health outcomes, such as usability, caregiver burden, and quality of life. Additional directions to advance native smartphone applications for caregivers are discussed, including personalization and expansion of eligibility criteria.

## Introduction

In Canada, individuals with chronic conditions represent approximately 44.2% of the adult population aged 20 years or older, and at least one in every five adults is estimated to have two or more chronic illnesses^1^. Informal caregivers, such as family or friends, may be able to offer assistance with basic activities of daily living (ADL), such as feeding and walking, as well as instrumental activities of daily living (IADL), such as preparing meals, managing medications, and transportation^2^. Caregiving has been associated with varied outcomes, which could be positive such as personal growth, or negative outcomes such as emotional exhaustion^3^. Previous research has found that individuals in a caregiver role were at greater risk of injuries and illnesses, such as anxiety and depression^4^. The progressive and complex nature of some chronic conditions can lead some individuals to experience caregiver burden^5^. In particular, the transition to a high-intensity caregiving role (i.e., providing support for ADL) was found to be associated with the functional decline of family caregivers^6^. People may experience burden differently based on the type and frequency of the caregiving, as well as their own perceptions toward care-related tasks or problems^5^. With the growing prevalence of chronic conditions in an aging population, more attention is needed to prepare and support individuals in a caregiving role^7,8^.

In the past decade, smartphones have become sophisticated and affordable computing devices, playing a key role in digital inclusion^9^. Smartphones can have multiple applications (referred to as native apps), some of which may enable users to access online resources, including web-based applications. One of the key advantages of native apps over web-based applications is that the former is installed on the user’s mobile device^10^. Thereby, some native apps can be used offline by individuals who live in underserved or rural locations with limited or no access to broadband Internet. Moreover, native apps can also gain access to built-in components (e.g., camera, global positioning system, and biometric sensors) and other external devices connected through Bluetooth or Wi-Fi. System designers can leverage these technologies to design context-aware native apps that are tailored to user preferences and/or environment.

Digital health technologies (DHT), including native and web-based applications, have been explored as a means to deliver health interventions for caregivers^11,12^. For example, a systematic review in 2018 investigated web-based applications targeting chronic conditions in general and found a low-to-moderate positive impact on caregivers’ self-esteem, self-efficacy, mastery, and strain^12^. Considering that different chronic conditions may present unique challenges for informal caregivers, some reviews have focused on single chronic illnesses, such as dementia^13,14^ or cancer^15^. Even though these previous reviews investigated a broad range of technologies, the literature on native apps dedicated to specific chronic conditions is scarce. A study of two international application stores identified several native apps targeting chronic conditions in general, but found no supporting evidence for these apps in the literature^16^. Ultimately, a siloed approach for the management of chronic conditions may lead to the concomitant adoption of multiple native apps and create repetitive or redundant tasks for caregivers^17^. With multiple chronic conditions (MCC) becoming more prevalent in an aging population, informal caregivers will need tailored apps to support individuals with two or more chronic conditions living in the community^18^.

The objective of this scoping review is to summarize emergent research on native apps exclusively designed to support informal caregivers across various common types of chronic conditions. The research question is: what is known in the literature about native apps for informal caregivers of chronically ill patients living in the community?

## Results

### Article characteristics

**Fig. 1:**
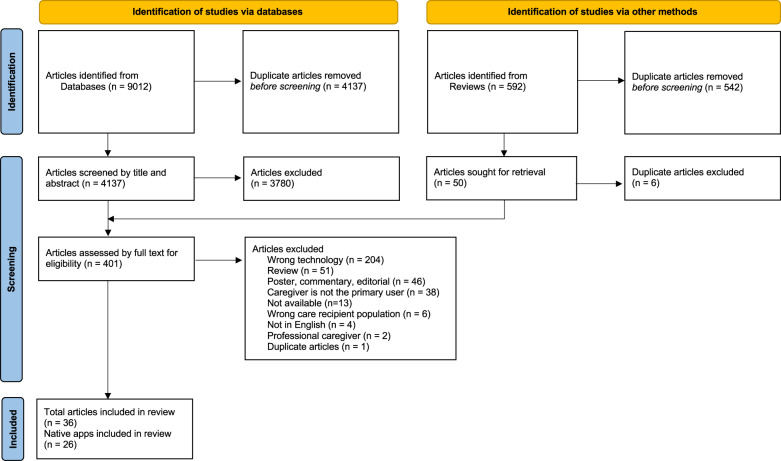
Flow diagram. Preferred reporting items for systematic review and meta-analysis (PRISMA) flow diagram of the literature search^19^.

Searches of six databases yielded a total of 9012 articles. After removing duplicates, there were 4137 articles that were screened by title and abstract, resulting in 357 articles that were eligible for full text screening. In addition, another 44 articles were identified through hand-searching the citations of retrieved reviews that were eligible for full-text screening, resulting in a total of 401 articles that were screened by full text. After full-text screening, four conference proceedings and 32 journal articles were included in the review, totaling 36 articles, encompassing 26 native apps. Most apps were associated with only one study (*n* = 18). The results of the article-selection process are shown in the Preferred Reporting Items for Systematic Review and Meta-Analysis (PRISMA)^19^ flow diagram in Fig. 1. Table 1 describes the main characteristics of included articles. Most studies originated from research groups in the United States (*n* = 12) and the Netherlands (*n* = 5) and were published within the past two years, in 2019–2020. Most articles were intended for caregivers of a person with Alzheimer’s or other dementia (*n* = 27) and only one study addressed two or more chronic illnesses (i.e., MCC). Included studies presented varied research designs, with case studies being the most common design (*n* = 14). Findings of effectiveness studies were available for 5 apps, out of which 3 were publicly accessible.

**Table 1:** Study characteristics.

Article characteristics	Number of articles out of *N* = 36	References
*Country of origin*^*a*^		
Australia	3	^39,43,62^
Austria	1	^33^
Brazil	1	^36^
Denmark	1	^49^
Germany	1	^56^
Greece	2	^40,60^
India	1	^41^
Ireland	1	^40^
Mexico	1	^42^
Norway	1	^38^
Pakistan	2	^57,63^
Poland	1	^49^
South Korea	1	^51^
Spain	2	^28,49^
Sweden	1	^58^
The Netherlands	5	^31,37,40,44,47^
Turkey	1	^45^
United Kingdom	2	^26,40^
United States	12	^27,32,34,35,46,48,50,52–55,59^
Not specified EU Country	1	^61^
*Publication year*		
2011	1	^42^
2014	1	^54^
2015	1	^55^
2016	4	^40,46,49,57^
2017	4	^31,47,48,56^
2018	3	^38,60,61^
2019	10	^27,32,34,36,37,39,41,43,44,50^
2020	10	^26,28,33,35,45,51,52,58,59,63^
2021	2	^53,62^
*Chronic illness*^a^		
Alzheimer’s or dementia	27	^26,27,31,33–38,42–44,46–56,58,60–62^
Anxiety or depression	2	^28,40^
Asthma	0	–
Cancer	4	^32,39,45,59^
Cerebrovascular disorders	3	^41,57,63^
COPD^b^	1	^40^
Diabetes	1	^40^
Heart failure	1	^40^
Hypertension	0	–
Rheumatoid arthritis	0	–
*Research design*		
Case study	14	^26,31–34,36,38,39,42,43,46,48,55,62^
Descriptive	4	^54,56,60,61^
Observational, cross-sectional	1	^41^
Phenomenology	1	^40^
Pretest/posttest	5	^28,45,50,51,58^
Randomized controlled trial	11	^27,35,37,44,47,49,52,53,57,59,63^

^a^Multi-site and multiple chronic conditions studies are counted in more than one category.

^b^COPD Chronic obstructive pulmonary diseases.

### Smartphone-application characteristics

In total, 26 native apps were identified in this review. Table 2 summarizes the main characteristics of the apps in terms of frequency of use, targeted platform(s), distribution, study type and year, categories of intended users, methods employed to build the app, and theoretical frameworks that informed app development. Additional information about the functionalities and/or system components of included apps can be found in Supplementary Table 2. The apps have been grouped by chronic condition, including Alzheimer’s and other dementia (*n* = 18), cancer (*n* = 4), stroke (*n* = 2), depression (*n* = 1), and MCC (*n* = 1). Most common components and/or system functionalities offered by native apps for caregivers include education about the chronic condition related to the patient’s care (*n* = 13), communication with family, friends, peers, or healthcare providers (*n* = 9), screening for risk factors (*n* = 7), self-care for the caregiver (*n* = 4), social networks (*n* = 3), personalized feedback (*n* = 3), and storing and/or sharing health information of the patient (*n* = 3), among others. The frequency of use refers to a recommended ‘dose’ of an intervention, which in the context of digital health interventions, is often related to how often participants are instructed to log in or perform specific tasks within the app. Owing to the diversity of approaches to measure the frequency of use, the usage was considered as either *fixed* (i.e., regular use of the app was suggested/requested, such as daily or weekly) or *as needed* (i.e., no frequency was defined for usage). Android was the most common platform (*n* = 18), and several native apps supported two or more platforms (*n* = 15), such as iOS and web platforms (i.e., the user can access the app from a browser, such as Safari or Google Chrome). Before 2016, most articles described private apps (i.e., apps that were only available to study participants or to external researchers upon request). After this date, many articles reported on public apps, which could be downloaded by the public from an app store such as Google Play (Google) or App Store (Apple).

**Table 2:** Characteristics of native smartphone applications for caregivers grouped by chronic condition (italicized).

Application name (Distribution)	Use frequency	Platform	Study type (Ref)	User	Method	Theoretical framework
*Alzheimer’s or dementia*						
C-MMD^a^(Private)	As needed	Not specified, web	Development^60,61^, Usability^26^	Caregiver, patient, provider	UCD	NR^b^
CareHeroes (Public)	As needed	Android, web	Feasibility^46^, Pilot^34^	Caregiver, provider	UCD	Family-centered theory^22^
CAST^c^ (Private)	Fixed	Android	Feasibility^48^	Caregiver	UCD	NR
Cubes (Public)	As needed	Android, iOS	Usability^31^	Caregiver, provider	UCD	NR
Dea (Private)	As needed	Android	Usability^33^	Caregiver	UCD	Meaningful Activity^79^
Dementia Support for Carers (Private)	As needed	Android, iOS	Protocol^43^, Development^62^	Caregiver	UCD	Family-centered theory^22^, empowerment model^80^, and adult learning theory^81^
FamTechCare (Public)	Fixed	iOS, web	Effectiveness^27,52^, Feasibility^35^, Cost-effectiveness^53^	Caregiver	NR	Reasons and management of behavioral and psychological symptoms of dementia^24^
Inlife (Public)	As needed	Android, iOS, web	Protocol^47^, Implementation^37^	Caregiver	UCD	NR
MemoryBoard (Private)	Fixed	Android, web	Usability^42^	Caregiver, patient	UCD	NR
MIT^d^(Public)	Fixed	iOS	Feasibility^50^	Caregiver	NR	Mentalization theory^22^
mYouTime (Private)	As needed	Android, iOS	Usability^38^	Caregiver, provider	UCD	NR
PsyMate (Public)	Fixed	Android, iOS	Effectiveness^44^	Caregiver	NR	Experience sampling method^82^
SMAI^e^(Private)	Fixed	Android, web	Usability^36^	Caregiver, provider	UCD	NR
Story-call (Private)	As needed	Android	Development^54^, Pilot^55^	Caregiver	NR	Resilience model of family stress, adjustment, and adaptation^23^
UnderstandAID (Private)	As needed	Android	Pilot^49^	Caregiver	NR	NR
Unnamed (Private)	As needed	Android	Usability^56^	Caregiver, patient	NR	NR
Unnamed (Private)	Fixed	Not specified	Effectiveness^51^	Caregiver	MADL	Reasons and Management of Behavioral and Psychological Symptoms of Dementia^24^
Unnamed (Private)	As needed	Not specified	Protocol^58^	Caregiver	UCD	NR
*Anxiety or depression*						
Happy (Public)	Fixed	Android, iOS	Pilot^28^	Caregiver	NR	NR
*Cancer*						
Caregiver Communication about Cancer (Public)	As needed	iOS	Acceptability^32^	Caregiver	NR	Family caregiver communication typology^83^
Carer Guide App (Public)	As needed	Android, iOS, web	Usability^39^	Caregiver	UCD	TPB^f^ ^25^ and UTAUT^g^ ^84^
Roadmap 2.0 (Public)	Fixed	Android, iOS	Protocol^59^	Caregiver, patient	UCD	NR
Unnamed (Public)	As needed	Android, iOS	Effectiveness^45^	Caregiver	NR	NR
*Cerebrovascular disorder*						
Movies4Stroke (Private)	Fixed	Android	Protocol^57^, Effectiveness^63^	Caregiver, patient	NR	Rogers’ diffusion of innovation theory^85^
Unnamed (Private)	NR	Not specified	Acceptability^41^	Caregiver, patient	NR	NR
*Multiple chronic conditions*						
WELCOME^h^(Private)	As needed	Not specified, web	Development^40^	Caregiver, patient, provider	UCD	NR

^a^C-MMD: CaregiversPro-MMD.

^b^NR: not reported.

^c^CAST: Caregiver Assessment Using Serious Gaming Technology.

^d^MIT: Mentalizing Imagery Therapy.

^e^SMAI: Mobile System for Elderly Monitoring.

^f^TPB: Theory of planned behavior.

^g^UTAUT: Unified theory of acceptance and use of technology.

^h^WELCOME: Wearable Sensing and Smart Cloud Computing for Integrated Care to COPD Patients with Comorbidities.

### Software design and development

The user-centered design (UCD) method (also referred to as human-centered design) was commonly cited as being used to develop native apps (*n* = 14), which meant that caregivers were engaged at different stages of software development to generate ideas or validate the function of the system^20^. Among the apps developed with UCD, several studies focused on improving usability (*n* = 6), as shown in Table 2. Usability studies were conducted on different versions of the app, ranging from paper-based prototypes in early formative research to functional apps in more advanced stages. Besides UCD, one study mentioned the mobile-application development lifecycle (MADL)^21^, derived from the waterfall method^10^. Most native apps were designed to be used only by caregivers (*n* = 15), with a few apps also targeting care recipients (*n* = 5), healthcare providers (*n* = 4), or all three roles (*n* = 2). Some of the articles referenced a theoretical framework when detailing the main features and/or components supported by the technology (*n* = 11). Frameworks used were specific to caregiving, such as the family-centered theory^22^ (*n* = 2) and the resiliency model of family stress, adjustment, and adaptation^23^ (*n* = 1), to the chronic condition, such as reasons and management of behavioral and psychological symptoms of dementia^24^ (*n* = 1), or to the intervention type, such as the theory of planned behavior^25^ (*n* = 1).

### Study participants

The criteria for selecting participants in research with native apps varied greatly among the included studies. Table 3 presents a nonexhaustive list of the most frequent criteria used for selecting caregiver study participants. The most common inclusion criterion was self-identification as a primary caregiver (*n* = 21), which was less restrictive than the requirement to have a relationship with the care recipient, such as a partner, sibling, or child (*n* = 9). Moreover, several articles had inclusion criteria based on language skills (written or spoken) (*n* = 11), age (*n* = 9), living arrangements (e.g., sharing the same household or living within a short-distance drive of the care recipient) (*n* = 8), access to the Internet (*n* = 8), and access to a computer or smartphone (*n* = 6). Articles reported exclusion criteria less frequently (*n* = 24). These criteria included health issues (*n* = 7), cognitive impairment (*n* = 4), and caregiver burden (*n* = 3). The criteria to exclude caregivers based on burden was generally not defined in absolute terms and, instead, was individually assessed by the research team. Some articles reported challenges in recruiting caregivers who were available and interested to participate in the evaluation of the native app^26–28^ and consequently relaxed the selection criteria to address these challenges. As characteristics of caregivers participating in research, several studies reported the gender, education, caregiving experience, caregiving intensity, working situation, ethnicity, and/or frequency of technology use by participants.

**Table 3:** Eligibility criteria of caregiver study participants.

Characteristic	Used as inclusion criteria	Used as exclusion criteria
Access to computer/smartphone	^28,32,33,50,51,58^	^41,45^
Access to the Internet	^37,43,46,47,51,54,55,58^	
Age	^26,32,34,39,43,45,50,58,59^	^45,58^
Caregiver burden	^49^	^37,44,47^
Caregiving experience	^49,51,58^	
Cognitive impairment		^44,49,57,63^
Familiarity with technology	^31,37,45,47,63^	^45^
Health issues	^28^	^28,37,44,47,49,51,58^
Language skills	^26,32,34,39,41,43,45,46,50,58,59^	
Literacy/education level	^45^	^45,49^
Living arrangements	^27,35,44,46,52,53,57,63^	
Relationship with care recipient	^27,28,31,35,36,38,44,49,56^	
Self-identify as caregiver	^26,32,33,37,39–41,43,45–48,50–55,57,59,63^	
Not reported/none	^42,60–62^	^26,27,31–36,38–40,42,43,46,48,52–56,59–62^

### Outcomes measured

According to the CONSORT-EHEALTH^29^ reporting guidelines, studies on DHT should describe use outcomes in addition to primary/secondary health outcomes. Use outcomes (e.g., engagement, frequency of use, or adherence) and nonuse outcomes (e.g., attrition) are examples of process outcomes required for the interpretation of results. Quality characteristics of the system, such as usability, effectiveness (i.e., accuracy and completeness with which users achieve specified goals), and efficiency (i.e., resources consumed to achieve goals)^30^, can facilitate process outcomes, such as adoption and acceptability. Overall, 14 articles investigated only process outcomes^26,31–43^; 11 articles investigated only health outcomes^27,44–53^; 7 articles investigated both^28,54–59^; and four articles did not investigate primary or secondary health outcomes for caregivers^60–63^.

The CONSORT-EHEALTH guidelines also recommend the use of qualitative and quantitative methods for a comprehensive evaluation of DHT; whereby qualitative methods help explore subjective perceptions of participants regarding the system, and quantitative methods (e.g., surveys and scales) provide an objective measure of an attribute and/or concept^29^. The majority of articles included in this review employed quantitative methods (*n* = 13)^26–28,41,44,45,49,51–53,56,57,63^ or a combination of quantitative and qualitative methods (*n* = 10)^32,36,37,39,42,46,47,50,58,59^. Table 4 lists the most common outcomes and associated quantitative instrument measures utilized in research, such as caregiver burden (*n* = 9), depression (*n* = 8), and quality of life (*n* = 4).

**Table 4:** Quantitative instruments used to explore caregiving, health/wellbeing, and process outcomes.

Outcome Group	Outcome(s)	Assessment instrument(s)	Ref
Caregiving	Burden, stress	Zarit Burden Interview (ZBI) (Custom*, 3-item)^48^	^48^
		Zarit Burden Interview (ZBI) Screening (4-item)^86^	^46,54,55^
		Zarit Burden Interview (ZBI) Short (12-item)^86^	^27,56,58^
		Zarit Burden Interview (ZBI) (22-item)^87^	^49,51^
Caregiving	Caregiver competence, sense of competence	Caregiver Competence (CCS) (Custom, 4-item)^88^	^49^
		Short Sense of Competence Questionnaire (SSCQ) (7-item)^89^	^27,44,47^
Caregiving	Quality of life, Care-related quality of life	CarerQoL (7-item)^90^	^47,58^
		PROMIS Global Health-10 scale^91^	^59^
		Quality of Life—Family Version (QoL-FV)^92^	^45^
Health/wellbeing	Anxiety, depression, depressive symptoms	Center for Epidemiological Studies Depression Scale (CES-D) (20-item)^93^	^27,28,44,49^
		Hospital Anxiety and Depression Scale (HADS) (7-item)^94^	^44,47^
		Patient Health Questionnaire-2 (PHQ-2) (2-items)^95^	^46^
		Patient Health Questionnaire-9 (PHQ-9) (9-items)^96^	^58^
		Quick Inventory of Depressive Symptoms-Self-Rated (QIDS) (16-item)^97^	^50^
Health/wellbeing	Social support, social support interactions, social support relations	Gain Through Group Involvement Scale (GAINSCL) (15-item)^98^	^54,55^
		Social Support List 12-Interactions (SSL12-I) (12-item)^99^	^47^
		Multidimensional Scale of Perceived Support (MSPSS) (12-item)^100^	^47^
Health/wellbeing	Stress	Perceived Stress Scale (PSS) (10-item)^101^	^44,47^
Process	Tool satisfaction, mobile application rating	Program Participation Questionnaire (PPQ) (Custom, 34-item)^102^	^37,47^
		User Version of Mobile App Rating Scale (uMARS) (20-item)^103^	^43^
Process	Usability	ISONORM 9241/10 (7-item)^104^	^56^
		System Usability Scale (10-item)^105^	^33,43^

*An instrument labeled as “custom” indicates that it was adapted and/or shortened to fit research purposes.

## Discussion

### Principal findings

Smartphones offer a new avenue to support informal caregivers of chronically ill individuals. Recent reviews of international application stores identified several native apps targeting caregivers^16^, yet the scholarly literature about the design and evaluation of native apps remains scarce^11^. Literature reviews on technologies developed for caregivers have mainly focused on single chronic conditions, such as dementia, and include general-purpose apps, such as social media and videoconferencing^64^. In contrast to previous reviews, we investigated native apps within a broader set of chronic conditions, including combinations thereof. Also, this scoping review incorporates literature from different disciplines, such as psychology, medicine, and computer science. Despite the broader focus of this review, most included articles were published in the last 2 years, indicating the nascency of this growing research area. The following discussion focuses on supports for various chronic conditions, design and evaluation of DHT for informal caregivers, and research considerations.

### Supports for chronic conditions

Much of the literature covered in this review consists of native apps for Alzheimer’s and other dementia (n = 18), many of which were exclusively designed for caregivers (*n* = 11) and presented components and/or features for the education of caregivers about dementia, socialization (e.g., support groups, social networks), and/or self-care (e.g., mindfulness, journaling). However, this review identified a lack of research on native apps among other prevalent chronic conditions for an adult population, such as COPD, arthritis, or diabetes, and only one app targeting MCC. It can be challenging to find appropriate guidance for two or more chronic conditions as caregivers may need to be in close communication with a wide variety of health care providers to understand the specific needs of care recipients^18^, which may include pain management, palliative care, and/or multiple medications (polypharmacy). Considering the high prevalence of MCC in the adult population, more research is needed to understand the supports required by caregivers for managing various comorbidities and, possibly, the extent to which existing apps for single chronic conditions may be appropriate for MCC.

### Design and evaluation considerations

Smartphone applications could play an important role for the democratization of healthcare, and many of the apps found in this review offer new opportunities for caregivers to access health information. In addition to providing health information, some apps aim to fulfill specific caregiving needs, such as planning activities with the care recipient (e.g., social visits, appointments), making medical decisions (e.g., as a surrogate or shared-decision maker), or building caregiving skills (e.g., coping strategies, communication, competence). Caregivers may need support in different areas, and the personalization of apps has been suggested to support specific caregiving needs^34^. In particular, the personalization of apps could help mitigate issues concerning the quantity and quality of general health information, such as information overload^18,65^ and poor readability^66^. Several features may be explored to personalize apps based on common, foreseeable needs of caregivers, such as filters that direct users to specific resources, algorithms that make recommendations based on usage data and/or preferences (e.g., age, language, or location), or adaptive technologies that improve accessibility for individuals with a range of characteristics and capabilities^67^, such as closed captioning, text-to-speech, and coloring^9^. To meet caregiving needs that may be specific and/or time-sensitive, some DHT may introduce components that are individually tailored ^27,42,44,52^.

Several apps found in this review aimed to reduce caregiver burden. A previous meta-analysis found no effect of web-based interventions on caregiver burden, possibly due to heterogeneous technologies and caregiver characteristics observed among studies^12^. In this review, different versions of the Zarit Burden Scale were selected to measure burden, resulting in variability that could also make it difficult to compare research findings^68^. More recently, an understanding of informal caregiver burnout has emerged in which subjective burden is considered a measure of appraisal of the caregiving experience rather than as an outcome^3^. The use of theoretical frameworks for the design and evaluation of DHT has been recommended as a means to clarify relationships between intervention components and primary and/or secondary health outcomes^69,70^. However, only a few native apps in this review refer to a theoretical framework that is specific to caregiving, such as the family-centered theory^22^. Considering both positive and negative outcomes as part of a caregiver’s lived experience is important for a more balanced view^3,71^. Therefore, a comprehensive theory about caregiving could be particularly beneficial because it could help explain variations in health outcomes.

### Research considerations

Considerations about the eligibility criteria and recruitment strategies of research participants are usually made early in the investigation of novel technologies and have implications for the interpretation and generalizability of research findings. Nonrecruitment and self-selection have been observed to limit the participation of vulnerable populations in research involving DHT^72^, and more research is needed to reach out to vulnerable caregivers who could be at greater risk of experiencing caregiving burden, such as those who dedicate several hours per day to caregiving, have low education, or face financial stress^73^. Some articles included in this scoping review have reported challenges to recruiting caregivers^26–28^ and the issue may have been compounded by strict selection criteria targeting individuals with specific abilities and/or material means, such as ownership of a smartphone of a particular brand, fluency in one language, or access to the Internet at home. Thereby, studies investigating DHT could have inadvertently excluded research participants within particularly important demographics^74^, such as caregivers who live in underserved or remote areas, who do not own smartphones, or who are cognitively impaired.

Rigorous research designs, such as randomized controlled trials, are an essential step in determining whether a DHT is able to achieve desired health outcomes^70,75,76^. This review identified a lack of studies aimed to provide evidence on the effectiveness of native apps for informal caregivers, with only five native apps investigated in clinical trials. For highly adaptable DHT in which research participants may be assigned different components and/or features (e.g., system notifications, frequency of use, or educational resources), complex research designs can be used to compare intervention variations^75^. Furthermore, considering that caregiving is an activity that may require substantial commitment over an extended period of time, long-term effects of using apps require further investigation^77^, including apps with custom-evaluative features to screen and/or follow-up on research participants.

### Study limitations

Limitations to this study include that the search strategy may have missed potentially relevant articles not indexed by the databases selected in this review. To help mitigate this issue, an expert librarian revised the search strategy, and the reference lists of retrieved reviews were hand-searched to identify additional articles. It is also possible that tablet applications were excluded if compatibility with smartphones was not explicitly stated in the article. This scoping review aims to provide a preliminary map of the literature on native apps for informal caregivers, including apps at very early stages of research and development. As a common limitation of scoping reviews^19^, this review does not include a critical appraisal of the methodological quality and risk of bias of the included articles.

## Conclusions

This scoping review explores emergent native apps aimed to support informal caregivers across a diverse set of chronic conditions. Most studies included in the scoping review target caregivers of individuals with Alzheimer’s and other dementia, but key application features and/or components (e.g., education, screening, and social support) could be useful for other caregiver groups as well. By investigating the design and development of apps across various common types of chronic illnesses, this review aims to support the development of DHT for those caring for individuals with MCC. Due to heterogeneous designs and methods employed in the evaluation of apps, as well as the scarce number of trials, limited evidence is currently available for meta-analysis of clinical effectiveness. Further research is needed to understand how DHT could benefit caregivers and care recipients and to personalize apps based on specific caregiving needs.

## Methods

### Design

This scoping review followed Arksey and O’Malley’s framework for performing scoping reviews^78^. The framework comprises of (i) identifying the research question; (ii) searching for relevant studies; (iii) selecting relevant studies; (iv) charting the data; and (v) collating, summarizing, and reporting the results. The search terms combined subject headings and text words related to the three concepts of caregivers, DHT, and chronic diseases. For the review, the ten major chronic diseases prevalent in Canada as identified by the Public Health Agency of Canada were used, which were namely heart disease, stroke, cancer, chronic obstructive respiratory disease (COPD), asthma, diabetes, Alzheimer’s disease or other dementia, rheumatoid arthritis, hypertension, and mood or anxiety disorders^1^. Search strategies were reviewed by an experienced biomedical librarian from the Gerstein Science Information Center, University of Toronto. All search strategies can be found in the the Supplementary Table 1. Relevant articles were searched in Medline, Embase, CINAHL, ProQuest, PsycINFO, and ACM Digital Library. References of retrieved reviews were hand-searched to identify additional relevant articles. The initial searches were conducted in December 2019 and subsequently repeated to capture additional articles indexed until January 2021.

### Selection criteria

Articles were included if they met all of the following inclusion criteria: (i) care recipients are adults aged 18 years or older who have been diagnosed with one or more of the 10 chronic diseases stated above; (ii) the article describes or evaluates a native app that can be installed in the caregiver’s smartphone; and (iii) the app was purposively developed with a primary goal to support informal caregivers (e.g., family or friends of the care recipient). Articles could encompass full-length journal articles and conference proceedings. Conversely, articles were excluded if they met any of the following exclusion criteria: (i) the app targets institutionalized care recipients (e.g., long-term care, nursing homes, and hospitalized) and/or professional caregivers (e.g., clinicians, nurses, or paid support workers); (ii) the study investigates a general-purpose technology (e.g., social media, videoconferencing, and messenger) that is not tailor-made for caregiving; (iii) the article is not in English; or (iv) the article is a review, perspective, opinion, fast abstract, or commentary.

### Selection of studies

Two researchers (MG and AS) independently applied the selection criteria to all articles retrieved through the search strategy in a two-phased process. First, the title and abstract of all entries in the dataset were screened in duplicate. Second, both reviewers screened the full text of the remaining entries to confirm their relevance to the research question. Disagreements on the selection of articles were resolved among the two reviewers in a consensus meeting. At this stage, one author (MG) hand-searched the reference list of retrieved reviews to identify additional articles not indexed by any of the databases searched in this review. The entire process was iterated once more when the database searches were repeated in January 2021. Mendeley (Elsevier) was used for managing references, Covidence (Veritas Health Innovation Ltd.) was used to support independent screening and data extraction, and Excel (Microsoft) was used for data analysis.

### Data extraction and data analysis

Two authors (MG and AS) independently extracted data from included articles. Based on the CONSORT-EHEALTH guidelines^29^, the following information from the included articles was collected: (i) article characteristics (i.e., author(s), publication year(s), and country of origin); (ii) caregiver characteristics (e.g., age, gender, and education); (iii) study characteristics (e.g., theoretical framework, research design, purpose, selection criteria, and outcomes); and (iv) application details (e.g., targeted chronic conditions, platforms, distribution, usage mode, and development stage). Once the information from all included articles was extracted, descriptive quantitative analysis was used to summarize the frequency and distribution of native apps among platforms, chronic conditions, study design, caregiver participant-selection criteria, and investigated outcomes. Finally, all authors met to collectively discuss the areas in which the design and development of native apps for caregivers could be improved based on the information from the included articles.

### Reporting summary

Further information on research design is available in the Nature Research Reporting Summary linked to this article.

## Supplementary information

Supplementary Information File

Reporting Summary

## Data Availability

The aggregate data extracted and analyzed for this scoping review are available from the corresponding author on reasonable request.
