# Supplementary material for: Smartphone applications for informal caregivers of chronically ill patients: a scoping review
Source: NPJ Digit Med. 2022 Mar 21;5:33. doi: 10.1038/s41746-022-00567-z (PMC8938465; doi:10.1038/s41746-022-00567-z)
Supplement: Supplementary file 1 — Supplementary Information File [file 41746_2022_567_MOESM1_ESM.pdf]

Supplementary Table 1. Search strategy used to retrieve studies from publication databases

Medline

1. Caregivers/
2. ((famil* or informal or partner? or spouse? or sibling? or child* or volunteer?) adj2 (caregiv* or care giv* or carer?)).tw,kf.
3. 1 or 2
4. Telemedicine/
5. Electronic Health Records/
6. Consumer Health Informatics/
7. Internet/
8. Mobile Applications/
9. (internet or health* informatics or technolog*).tw,kf.
10. health records, personal/ or patient generated health data/ or patient portals/
11. (telemonitor* or tele monitor* or telecare or tele care or telehealth or tele health or telemedicine or tele medicine).tw,kf.
12. ((mobile or wearable or portable) adj4 (system? or device? or app* or tool?)).tw,kf.
13. (mhealth or m health or ehealth or e health).tw,kf.
14. or/4-13
15. chronic disease/ or multiple chronic conditions/ or noncommunicable diseases/
16. diabetes mellitus/ or diabetes mellitus, type 1/ or diabetes mellitus, type 2/
17. Pulmonary Disease, Chronic Obstructive/
18. Asthma/
19. exp Heart Diseases/
20. hypertension/
21. exp Cerebrovascular Disorders/
22. Arthritis, Rheumatoid/
23. (rheumatoid arthritis or polyarthritis).tw,kf.
24. Alzheimer Disease/
25. Neoplasms/
26. Major Depressive Disorder/
27. Anxiety Disorders/
28. ((anxiety or mood or depress* or neurotic) adj2 disorder?).tw,kf.
29. (chronic* adj2 (ill* or disease? or disorder? or condition?)).tw,kf.
30. ((noncommunicable or non communicable) adj2 (disease? or disorder? or condition?)).tw,kf.
31. (COPD or ((pulmonary or respiratory or lung) adj2 (disease? or disorder?))).tw,kf.
32. (diabetes mellitus or (diabetes adj2 (type 1 or type 2)) or asthma*).tw,kf.
33. (hypertens* or high blood pressure).tw,kf.
34. ((heart or cardiac or cardiovascular or coronary) adj2 (stroke? or infarction or disease? or disorder? or failure or arrythmia?)).tw,kf.
35. ((cerebrovascular or vascular or carotoid$ or cerebral or brain) adj2 (stroke? or infarction or ischemia? or disorder? or disease?)).tw,kf.
36. (alzheimer* or dementia?).tw,kf.
37. (malignant neoplasm? or neoplas* or cancer* or tumor? or tumour?).tw,kf.
38. or/15-37
39. 3 and 14 and 38

Supplementary Table 1. (continued)

EMBASE

1. caregiver/
2. ((famil* or informal or partner? or spouse? or sibling? or child* or volunteer?) adj2 (caregiv* or care giv* or carer?)).tw,kw
3. 1 or 2
4. telemedicine/ or telehealth/ or telemonitoring/
5. electronic health record/
6. consumer health informatics/
7. Internet/
8. mobile application/
9. (internet or health* informatics or technolog*).tw,kw
10. electronic patient record/
11. (telemonitor* or tele monitor* or telecare or tele care or telehealth or tele health or telemedicine or tele medicine).tw,kw.
12. ((mobile or wearable or portable) adj4 (system? or device? or app* or tool?)).tw,kw
13. (mhealth or m health or ehealth or e health).tw,kw
14. or/4-13
15. chronic disease/ or multiple chronic conditions/ or non communicable disease/
16. diabetes mellitus/ or insulin dependent diabetes mellitus/ or non insulin dependent diabetes mellitus/
17. chronic obstructive lung disease/
18. asthma/ or mild intermittent asthma/ or mild persistent asthma/ or moderate persistent asthma/ or nocturnal asthma/ or severe persistent asthma/"
19. exp heart disease/
20. elevated blood pressure/ or hypertension/
21. exp cerebrovascular disease/
22. rheumatoid arthritis/
23. (rheumatoid arthritis or polyarthritis).tw,kw.
24. alzheimer disease/
25. neoplasm/ or malignant neoplasm/
26. major depression/
27. anxiety disorder/
28. ((anxiety or mood or depress* or neurotic) adj2 disorder?).tw,kw
29. (chronic* adj2 (ill* or disease? or disorder? or condition?)).tw,kw.
30. ((noncommunicable or non communicable) adj2 (disease? or disorder? or condition?)).tw,kw
31. (COPD or ((pulmonary or respiratory or lung) adj2 (disease? or disorder?))).tw,kw
32. (diabetes mellitus or (diabetes adj2 (type 1 or type 2)) or asthma*).tw,kw.
33. (hypertens* or high blood pressure).tw,kw.
34. ((heart or cardiac or cardiovascular or coronary) adj2 (stroke? or infarction or disease? or disorder? or failure or arrythmia?)).tw,kw
35. ((cerebrovascular or vascular or carotoid$ or cerebral or brain) adj2 (stroke? or infarction or ischemia? or disorder? or disease?)).tw,kw.
36. (alzheimer* or dementia?).tw,kw.
37. (malignant neoplasm? or neoplas* or cancer* or tumor? or tumour?).tw,kw
38. or/15-37
39. 3 and 14 and 38

Supplementary Table 1. (continued)

PsycINFO

1. caregivers/
2. ((famil* or informal or partner? or spouse? or sibling? or child* or volunteer?) adj2 (caregiv* or care giv* or carer?)).ti,ab
3. 1 or 2
4. telemedicine/ or electronic health services/ or mobile health/
5. electronic health records/
6. internet/
7. mobile devices/ or smartphones/ or mobile technology/ or mobile applications/ or wearable devices/
8. (internet or health* informatics or technolog*).ti,ab
9. client records/
10. (telemonitor* or tele monitor* or telecare or tele care or telehealth or tele health or telemedicine or tele medicine).ti,ab
11. ((mobile or wearable or portable) adj4 (system? or device? or app* or tool?)).ti,ab
12. (mhealth or m health or ehealth or e health).ti,ab
13. or/4-12
14. chronic illness/ or comorbidity/
15. diabetes mellitus/ or type 2 diabetes/
16. Chronic Obstructive Pulmonary Disease/
17. asthma/
18. exp heart disorders/
19. hypertension/
20. exp cerebrovascular disorders/
21. rheumatoid arthritis/
22. (rheumatoid arthritis or polyarthritis).ti,ab
23. alzheimer's disease/
24. neoplasms/
25. major depression/
26. anxiety disorders/ or generalized anxiety disorder/
27. ((anxiety or mood or depress* or neurotic) adj2 disorder?).ti,ab
28. (chronic* adj2 (ill* or disease? or disorder? or condition?)).ti,ab
29. ((noncommunicable or non communicable) adj2 (disease? or disorder? or condition?)).ti,ab
30. (COPD or ((pulmonary or respiratory or lung) adj2 (disease? or disorder?))).ti,ab
31. (diabetes mellitus or (diabetes adj2 (type 1 or type 2)) or asthma*).ti,ab
32. (hypertens* or high blood pressure).ti,ab
33. ((heart or cardiac or cardiovascular or coronary) adj2 (stroke? or infarction or disease? or disorder? or failure or arrythmia?)).ti,ab
34. ((cerebrovascular or vascular or carotoid$ or cerebral or brain) adj2 (stroke? or infarction or ischemia? or disorder? or disease?)).ti,ab
35. (alzheimer* or dementia?).ti,ab
36. (malignant neoplasm? or neoplas* or cancer* or tumor? or tumour?).ti,ab
37. or/14-36
38. 3 and 13 and 37

Supplementary Table 1. (continued)

CINAHL (EBSCO)

S1. (MH "Caregivers")
S2. TI((famil* OR informal OR partner# OR spouse# OR sibling# OR child* OR volunteer#) N2 (caregiv* OR "care giv*" OR carer#)) OR AB((famil* OR informal OR partner# OR spouse# OR sibling# OR child* OR volunteer#) N2 (caregiv* OR "care giv*" OR carer#))
S3. S1 OR S2
S4. (MH "Telemedicine") OR (MH "Telehealth")
S5. (MH "Electronic Health Records") OR (MH "Patient Portals")
S6. (MH "Internet")
S7. (MH "Mobile Applications") OR (MH "Telecommunications+")
S8. TI(internet OR "health* informatics" OR technolog*) OR AB(internet OR "health* informatics" OR technolog*)
S9. (MH "Medical Records, Personal")
S10. TI(telemonitor* OR "tele monitor*" OR telecare OR "tele care" OR telehealth OR "tele health" OR telemedicine OR "tele medicine") OR AB(telemonitor* OR "tele monitor*" OR telecare OR "tele care" OR telehealth OR "tele health" OR telemedicine OR "tele medicine")
S11. TI((mobile OR wearable OR portable) N4 (system# OR device# OR app* OR tool#)) OR AB ((mobile OR wearable OR portable) N4 (system# OR device# OR app* OR tool#))
S12. TI(mhealth OR "m health" or ehealth OR "e health") OR AB(mhealth OR "m health" or ehealth OR "e health")
S13. S4 OR S5 OR S6 OR S7 OR S8 OR S9 OR S10 OR S11 OR S12
S14. (MH "Chronic Disease") OR (MH "Noncommunicable Diseases")
S15. (MH "Diabetes Mellitus, Type 1") OR (MH "Diabetes Mellitus, Type 2") OR (MH "Diabetes Mellitus")
S16. (MH "Pulmonary Disease, Chronic Obstructive")
S17. (MH "Asthma")
S18. (MH "Heart Diseases+")
S19. (MH "Hypertension")
S20. (MH "Cerebrovascular Disorders+")
S21. (MH "Arthritis, Rheumatoid")
S22. TI ("rheumatoid arthritis" OR polyarthritis) OR AB ("rheumatoid arthritis" OR polyarthritis)
S23. (MH "Alzheimer's Disease")
S24. (MH "Neoplasms")
S25. (MH "Depression")
S26. (MH "Anxiety Disorders") OR (MH "Generalized Anxiety Disorder")
S27. TI((anxiety OR mood OR depress* OR neurotic) N2 disorder#) OR AB((anxiety OR mood OR depress* OR neurotic) N2 disorder#)
S28. TI(chronic* N2 (ill* or disease# or disorder# or condition#)) OR AB(chronic* N2 (ill* or disease# or disorder# or condition#))
S29. TI((noncommunicable or "non communicable") N2 (disease# or disorder# or condition#)) OR AB((noncommunicable or "non communicable") N2 (disease# or disorder# or condition#))
S30. TI(COPD OR ((pulmonary OR respiratory OR lung) N2 (disease# OR disorder#))) OR AB(COPD OR ((pulmonary OR respiratory OR lung) N2 (disease# OR disorder#)))
S31. TI("diabetes mellitus" OR (diabetes N2 ("type 1" OR "type 2")) or asthma*) OR AB("diabetes mellitus" OR (diabetes N2 ("type 1" OR "type 2")) or asthma*)
S32. TI (hypertens* OR "high blood pressure") OR AB (hypertens* OR "high blood pressure")
S33. TI((heart OR cardiac OR cardiovascular OR coronary) N2 (stroke# OR infarction OR disease# OR disorder# OR failure OR arrythmia#)) OR AB((heart OR cardiac OR cardiovascular OR coronary) N2 (stroke# OR infarction OR disease# OR disorder# OR failure OR arrythmia#))
S34. TI((cerebrovascular OR vascular OR carotoid* OR cerebral OR brain) N2 (stroke# OR infarction OR ischemia# OR disorder# OR disease#)) OR AB((cerebrovascular OR vascular OR carotoid* OR cerebral OR brain) N2 (stroke# OR infarction OR ischemia# OR disorder# OR disease#))
S35. TI(alzheimer* OR dementia#) OR AB(alzheimer* OR dementia#)
S36. TI("malignant neoplasm#" OR neoplas* OR cancer* OR tumor# OR tumour#) OR AB("malignant neoplasm#" OR neoplas* OR cancer* OR tumor# OR tumour#)
S37. S14 OR S15 OR S16 OR S17 OR S18 OR S19 OR S20 OR S21 OR S22 OR S23 OR S24 OR S25 OR S26 OR S27 OR S28 OR S29 OR S30 OR S31 OR S32 OR S33 OR S34 OR S35
S38. S3 AND S13 AND S37

Supplementary Table 1. (continued)

ProQuest

1. MESH.EXACT("Caregivers") OR MAINSUBJECT.EXACT("Caregivers")
2. TI((famil* OR informal OR partner* OR spouse* OR sibling* OR child* OR volunteer*) N/2 (caregiv* OR "care giv*" OR carer*)) OR AB ((famil* OR informal OR partner* OR spouse* OR sibling* OR child* OR volunteer*) N/2 (caregiv* OR "care giv*" OR carer*))
3. S1 OR S2
4. MESH.EXACT("Telemedicine") OR MAINSUBJECT.EXACT("Telemedicine")
5. MESH.EXACT("Electronic Health Records") OR MAINSUBJECT.EXACT("Electronic Health Records")
6. MESH.EXACT("Internet") OR MAINSUBJECT.EXACT("Internet")
7. MESH.EXACT("Electronic Technology") OR MAINSUBJECT.EXACT("Electronic Technology")
8. TI(internet OR "health* informatics" OR technolog*) OR AB(internet OR "health* informatics" OR technolog*)
9. MESH.EXACT("Medical Records") OR MAINSUBJECT.EXACT("Medical Records")
10. TI(telemonitor* OR "tele monitor*" OR telecare OR "tele care" OR telehealth OR "tele health" OR telemedicine OR "tele medicine") OR AB(telemonitor* OR "tele monitor*" OR telecare OR "tele care" OR telehealth OR "tele health" OR telemedicine OR "tele medicine")
11. TI((mobile OR wearable OR portable) N/4 (system* OR device* OR app* OR tool*)) OR AB ((mobile OR wearable OR portable) N/4 (system* OR device* OR app* OR tool*))
12. TI(mhealth OR "m health" OR ehealth OR "e health") OR AB(mhealth OR "m health" OR ehealth OR "e health")
13. S4 OR S5 OR S6 OR S7 OR S8 OR S9 OR S10 OR S11 OR S12
14. MESH.EXACT("Chronic Disease") OR MAINSUBJECT.EXACT("Chronic Illness")
15. MESH.EXACT("Diabetes Mellitus") OR MAINSUBJECT.EXACT("Diabetes")
16. MESH.EXACT("Pulmonary Disease, Chronic Obstructive")
17. MESH.EXACT("Asthma") OR MAINSUBJECT.EXACT("Asthma")
18. MESH.EXACT("Heart Diseases") OR MAINSUBJECT.EXACT.EXPLODE("Heart Diseases")
19. MESH.EXACT("Hypertension") OR MAINSUBJECT.EXACT.EXPLODE("Blood Pressure")
20. MESH.EXACT("Cerebrovascular Disorders")
21. MESH.EXACT("Rheumatoid Arthritis") OR MAINSUBJECT.EXACT("Arthritis")
22. TI ("rheumatoid arthritis" OR polyarthritis) OR AB ("rheumatoid arthritis" OR polyarthritis)
23. MESH.EXACT("Alzheimer Disease") OR MAINSUBJECT.EXACT("Alzheimer's Disease")
24. MESH.EXACT("Neoplasms") OR MAINSUBJECT.EXACT("Cancer")
25. MESH.EXACT("Depressive Disorder, Major") OR MAINSUBJECT.EXACT("Depression (Psychology)")
26. MESH.EXACT("Anxiety Disorder") OR MAINSUBJECT.EXACT("Anxiety")
27. TI((anxiety OR mood OR depress* OR neurotic) N/2 disorder*) OR AB((anxiety OR mood OR depress* OR neurotic) N/2 disorder*)
28. TI(chronic* N/2 (ill* OR disease* OR disorder* OR condition#)) OR AB(chronic* N/2 (ill* OR disease* OR disorder* OR condition*))
29. TI((noncommunicable OR "non communicable") N/2 (disease* OR disorder* OR condition*)) OR AB((noncommunicable OR "non communicable") N/2 (disease* OR disorder* OR condition*))
30. TI(COPD OR ((pulmonary OR respiratory OR lung) N/2 (disease* OR disorder*))) OR AB(COPD OR ((pulmonary OR respiratory OR lung) N/2 (disease* OR disorder*)))
31. TI("diabetes mellitus" OR (diabetes N/2 ("type 1" OR "type 2")) or asthma*) OR AB("diabetes mellitus" OR (diabetes N/2 ("type 1" OR "type 2")) or asthma*)
32. TI (hypertens* OR "high blood pressure") OR AB (hypertens* OR "high blood pressure")
33. TI((heart OR cardiac OR cardiovascular OR coronary) N/2 (stroke* OR infarction OR disease* OR disorder* OR failure OR arrythmia*)) OR AB((heart OR cardiac OR cardiovascular OR coronary) N/2 (stroke* OR infarction OR disease* OR disorder* OR failure OR arrythmia*))
34. TI((cerebrovascular OR vascular OR carotoid* OR cerebral OR brain) N/2 (stroke* OR infarction OR ischemia* OR disorder* OR disease*)) OR AB((cerebrovascular OR vascular OR carotoid* OR cerebral OR brain) N/2 (stroke* OR infarction OR ischemia* OR disorder* OR disease*))
35. TI(alzheimer* OR dementia*) OR AB(alzheimer* OR dementia*)
36. TI("malignant neoplasm*" OR neoplas* OR cancer* OR tumor* OR tumour*) OR AB("malignant neoplasm*" OR neoplas* OR cancer* OR tumor* OR tumour*)
37. S14 OR S15 OR S16 OR S17 OR S18 OR S19 OR S20 OR S21 OR S22 OR S23 OR S24 OR S25 OR S26 OR S27 OR S28 OR S29 OR S30 OR S31 OR S32 OR S33 OR S34 OR S36
38. S3 AND S13 AND S37

Supplementary Table 1. (continued)

ACM Digital Library
recordAbstract:(+(famil* informal partner* spouse* sibling* child* volunteer* +(caregiv* OR "care giver" OR "care givers" OR carer*)) +("electronic health record*" "patient portal*" "consumer health informatics" (health healthcare +(informatics technolog*)) "personal health record*" "electronic patient record*" tele*monitor* tele*care tele*health tele*medicine (mobile wearable portable +(device* application* app* tool*)) mhealth "m health" ehealth "e health" "mobile health") +("rheumatoid arthritis" polyarthritis "alzheimer disease" "alzheimer's disease" dementia* "major depressi*" (depress* anxiety mood neurotic +(disorder* disease*)) (chronic* +(disease* ill* condition conditions disorder*)) ("non communicable" noncommunicable +(disease* disorder* condition conditions)) COPD "chronic obstructive pulmonary disease" "chronic obstructive lung disease" (pulmonary respiratory lung +(disease* disorder*)) (diabetes +(mellitus "type 1" "type 2")) asthma* hypertension "high blood pressure" "elevated blood pressure" (heart cardiac cardiovascular coronary +(stroke* infarction disease* disorder* failure arrythmia*)) (cerebrovascular vascular carotoid cerebral brain +(stroke infarction ischemia* disease* disorder*)) "malignant neoplasm" neoplas* cancer* tumor* tumour*))

Supplementary Table 2. Description of native smartphone applications for caregivers

Application Name (Ref)	Description
Alzheimer's or dementia	
C-MMD [26,60,61]	A social network platform for persons with dementia, caregivers, and health care providers. It supports screening caregivers and care recipients for risk factors, cognitive training (games), sharing medical information, and tracking medication adherence
CareHeroes [34,46]	A social network platform for caregivers and health care providers. It enables self-assessments of depression and burden. It offers general information, linkage to external services, emotional support, and decision support regarding medications and challenging behaviors
CAST[b] [48]	A gaming app to measure and monitor stress and cognitive function of caregivers on a daily basis
Cubes [31]	A generic smartphone platform with several native apps for caregivers and health care providers. It combines independent apps for the caregiver education, health records of the care recipient, medication calendar, shared caregiving activities/events, direct communication (videoconferencing), and entertainment
Dea [33]	A recommendation system to promote activities with the care recipient. Content is presented as text and video. The tool also provides links to external resources (e.g., service facilities and self-help groups) and supports activity planning (events and exercises) and communication with peers
Dementia Support for Carers [43,62]	An educational platform for caregivers to manage functional disability (basic and instrumental activities of daily living)
FamTechCare [27,35,52,53]	A video-recording app that is activated by the caregiver to capture challenging behaviors of the care recipient and forward the recording to a health care provider. Videos are evaluated by a specialist team who communicates with the caregiver to offer personalized feedback on a weekly basis
Inlife [37,47]	A social network platform for caregivers. Individuals can join the circle of care of a person with dementia to receive updates, share health care information, and organize caregiving and social activities with the group
MemoryBoard [42]	A communication platform for caregivers and persons with dementia to automatically handle the repetitive questioning behavior of the care recipient. It supports setting personalized reminders and sharing calendar events on a daily basis
MIT [c] [50]	A therapy app for caregivers (self-care). Pre-recorded mentalizing practices are intended to be played every day. The app also advises caregivers on how to use the technique with the care recipient
mYouTime [38]	A video-sharing platform for caregivers and health care providers of persons with dementia. Content is created by trusted parties (e.g., health care providers) and can be enriched by text, hyperlinks, and subtitles
PsyMate [44]	A digital diary for caregivers (self-care). Enables self-assessments about mood and context (e.g., caregiving activities, social interactions, etc.) for three consecutive days a week. Caregivers receive personalized feedback from a specialist
SMAI[d] [36]	A telemonitoring app for persons with dementia managed by caregivers and health care providers. It supports remote monitoring of physiological parameters, medication adherence, and communication with health care providers on a daily basis.
Story-call [54,55]	A video-sharing platform for caregivers. Content is created by caregivers and validated by health care providers before sharing with members of the community
UnderstandAID [49]	An educational platform about dementia for caregivers. Topics are organized by complexity level (easy to complex). It enables the creation of calendar events or reminders for tasks such as medical appointments and medications. The app also offers a social network that is moderated by researchers
Unnamed [56]	A localization system for the persons with dementia that is remotely monitored by the caregiver. It automatically alerts the caregiver when the care recipient leaves a predefined area and enables the care recipient to place emergency calls
Unnamed [51]	An informational system covering topics about dementia, interventions (medications, environment, etc.), communication skills, coping methods, and bulletin boards (questions, news, and events). Caregivers also received weekly reminders to access the app.
Unnamed [58]	A professional (nurse) and peer support app with features that include messenger, journaling, mindfulness exercises, and events. Caregivers can also perform self-assessments

Supplementary Table 2. (continued)

Application Name (Ref)	Description
Anxiety or depression	
Happy [28]	An online support group system with information about depression, self-reinforcement, and mood. Information is organized into five different modules with self-guided exercises, including mood assessments. Caregivers were asked to complete one module per week
Cancer	
Caregiver Communication about Cancer [32]	An educational platform to increase communication skills of caregivers with health care providers and other family members and to learn about the disease and its management. The tool suggests talking points and allows the user to create to-do lists
Carer Guide App [39]	An informational platform for caregivers. It provides information about the disease and its daily management, linking caregivers to local health care resources. The tool will also have access to the user's contact list and will be integrated into a third-party social media platform (Facebook) to support communication
Roadmap 2.0 [59]	A recommendation system to promote daily positive practices, such as expressing gratitude or scheduling activities that promote positive thoughts, emotions, or behaviors (self-care).
Unnamed [45]	An educational platform to explain the radiotherapy process to families and patients. The tool includes videos, activities, and question-and-answer modules. The app includes symptom management information, relaxation exercises for the informal caregiver (self-care), and direct communication with the healthcare team (answered within 48hrs)
Cerebrovascular disorder	
Movies4Stroke [57,63]	An educational platform for caregivers and stroke survivors. It offers information on medications, worsening symptoms, and physical exercises to aid patient rehabilitation. After playing each video for the first time, caregivers complete a test to identify gaps in knowledge that can be individually addressed by an interventionist
Unnamed [41]	An app for physical activity targeting caregivers and stroke survivors. It offers pre-recorded home exercise lessons along with information about stroke recovery and rehabilitation. Caregivers also receive individualized feedback after each session
Multiple chronic conditions	
WELCOME[e] [40]	A telemonitoring app for people with COPD and heart failure, diabetes, anxiety, or depression as co-morbidities. Monitors physiological parameters (e.g., weight, blood glucose, blood pressure, heart rate, temperature, inhaler, mood, and smoking habits) as needed. Supports communication with health care providers and mental health assessments

[a] C-MMD: CaregiversPro-MMD, [b]CAST: Caregiver Assessment Using Serious Gaming Technology, [c] MIT: Mentalizing Imagery Therapy, [d] SMAI: Mobile System for Elderly Monitoring, [e] WELCOME: Wearable Sensing and Smart Cloud Computing for Integrated Care to COPD Patients with Co-morbidities
