# Supplementary material for: Smartphone applications for informal caregivers of chronically ill patients: a scoping review
Source: NPJ Digit Med. 2022 Mar 21;5:33. doi: 10.1038/s41746-022-00567-z (PMC8938465; doi:10.1038/s41746-022-00567-z)
Supplement: Supplementary file 2 — Reporting Summary [file 41746_2022_567_MOESM2_ESM.pdf]

nature portfolio

Corresponding author(s): Margarido,M.

Last updated by author(s): Dec 6, 2021

# Reporting Summary

Nature Portfolio wishes to improve the reproducibility of the work that we publish. This form provides structure for consistency and transparency in reporting. For further information on Nature Portfolio policies, see our Editorial Policies and the Editorial Policy Checklist.

## Statistics

For all statistical analyses, confirm that the following items are present in the figure legend, table legend, main text, or Methods section.

| n/a | Confirmed | |
|---|---|---|
| ☒ | ☐ | The exact sample size (*n*) for each experimental group/condition, given as a discrete number and unit of measurement |
| ☒ | ☐ | A statement on whether measurements were taken from distinct samples or whether the same sample was measured repeatedly |
| ☒ | ☐ | The statistical test(s) used AND whether they are one- or two-sided<br>*Only common tests should be described solely by name; describe more complex techniques in the Methods section.* |
| ☒ | ☐ | A description of all covariates tested |
| ☒ | ☐ | A description of any assumptions or corrections, such as tests of normality and adjustment for multiple comparisons |
| ☒ | ☐ | A full description of the statistical parameters including central tendency (e.g. means) or other basic estimates (e.g. regression coefficient) AND variation (e.g. standard deviation) or associated estimates of uncertainty (e.g. confidence intervals) |
| ☒ | ☐ | For null hypothesis testing, the test statistic (e.g. *F*, *t*, *r*) with confidence intervals, effect sizes, degrees of freedom and *P* value noted<br>*Give P values as exact values whenever suitable.* |
| ☒ | ☐ | For Bayesian analysis, information on the choice of priors and Markov chain Monte Carlo settings |
| ☒ | ☐ | For hierarchical and complex designs, identification of the appropriate level for tests and full reporting of outcomes |
| ☒ | ☐ | Estimates of effect sizes (e.g. Cohen's *d*, Pearson's *r*), indicating how they were calculated |

*Our web collection on statistics for biologists contains articles on many of the points above.*

## Software and code

Policy information about availability of computer code

| | |
|---|---|
| Data collection | Relevant articles were searched in Medline, Embase, CINAHL, ProQuest, PsycINFO, and ACM Digital Library. All search strategies can be found in the supplementary information file (see Supplementary Table 1). |
| Data analysis | Mendeley (Elsevier) was used for managing references, Covidence (Veritas Health Innovation Ltd.) was used to support independent screening and data extraction, and Excel (Microsoft) was used for data analysis. |

For manuscripts utilizing custom algorithms or software that are central to the research but not yet described in published literature, software must be made available to editors and reviewers. We strongly encourage code deposition in a community repository (e.g. GitHub). See the Nature Portfolio guidelines for submitting code & software for further information.

## Data

Policy information about availability of data

All manuscripts must include a data availability statement. This statement should provide the following information, where applicable:

- Accession codes, unique identifiers, or web links for publicly available datasets
- A description of any restrictions on data availability
- For clinical datasets or third party data, please ensure that the statement adheres to our policy

Data sharing not applicable to this article as no datasets were generated or analyzed during the current study.

# Field-specific reporting

Please select the one below that is the best fit for your research. If you are not sure, read the appropriate sections before making your selection.

☒ Life sciences ☐ Behavioural & social sciences ☐ Ecological, evolutionary & environmental sciences

For a reference copy of the document with all sections, see nature.com/documents/nr-reporting-summary-flat.pdf

# Life sciences study design

All studies must disclose on these points even when the disclosure is negative.

| | |
|---|---|
| Sample size | Not applicable to this study. |
| Data exclusions | Articles were excluded from the analysis if they met any of the following exclusion criteria: (i) the app targets institutionalized care recipients (e.g., long-term care, nursing homes, hospitalized) and/or professional caregivers (e.g., clinicians, nurses, or paid support workers); (ii) the study investigates a general-purpose technology (e.g., social media, videoconferencing, messenger, etc.) that is not tailor-made for caregiving; (iii) the article is not in English; or (iv) the article is a review, perspective, opinion, fast abstract, or commentary. |
| Replication | This scoping review followed Arksey and O'Malley's framework for performing scoping reviews. The methods section details the selection criteria and data extraction procedures required for replication of the search strategy. |
| Randomization | Not applicable to this study. |
| Blinding | Two researchers (MG and AS) independently applied the selection criteria to all articles retrieved through the search strategy in a two-phased process. Disagreements on the selection of articles were resolved among the two researchers in a consensus meeting. Then, the researchers proceeded to independently extract data from included articles. All authors met to collectively discuss the areas in which the design and development of native apps for caregivers could be improved based on the information from the included articles. |

# Reporting for specific materials, systems and methods

We require information from authors about some types of materials, experimental systems and methods used in many studies. Here, indicate whether each material, system or method listed is relevant to your study. If you are not sure if a list item applies to your research, read the appropriate section before selecting a response.

## Materials & experimental systems

| n/a | Involved in the study | |
|---|---|---|
| ☒ | ☐ | Antibodies |
| ☒ | ☐ | Eukaryotic cell lines |
| ☒ | ☐ | Palaeontology and archaeology |
| ☒ | ☐ | Animals and other organisms |
| ☒ | ☐ | Human research participants |
| ☒ | ☐ | Clinical data |
| ☒ | ☐ | Dual use research of concern |

## Methods

| n/a | Involved in the study | |
|---|---|---|
| ☒ | ☐ | ChIP-seq |
| ☒ | ☐ | Flow cytometry |
| ☒ | ☐ | MRI-based neuroimaging |
